# FLIMX: A Software Package to Determine and Analyze the Fluorescence Lifetime in Time-Resolved Fluorescence Data from the Human Eye

**DOI:** 10.1371/journal.pone.0131640

**Published:** 2015-07-20

**Authors:** Matthias Klemm, Dietrich Schweitzer, Sven Peters, Lydia Sauer, Martin Hammer, Jens Haueisen

**Affiliations:** 1 Institute of Biomedical Engineering and Informatics, Technische Universität Ilmenau, POB 100565, 98694, Ilmenau, Germany; 2 Experimental Ophthalmology, University Hospital Jena, Bachstraße 18, 07740, Jena, Germany; University of California, Berkeley, UNITED STATES

## Abstract

Fluorescence lifetime imaging ophthalmoscopy (FLIO) is a new technique for measuring the in vivo autofluorescence intensity decays generated by endogenous fluorophores in the ocular fundus. Here, we present a software package called FLIM eXplorer (FLIMX) for analyzing FLIO data. Specifically, we introduce a new adaptive binning approach as an optimal tradeoff between the spatial resolution and the number of photons required per pixel. We also expand existing decay models (multi-exponential, stretched exponential, spectral global analysis, incomplete decay) to account for the layered structure of the eye and present a method to correct for the influence of the crystalline lens fluorescence on the retina fluorescence. Subsequently, the Holm-Bonferroni method is applied to FLIO measurements to allow for group comparisons between patients and controls on the basis of fluorescence lifetime parameters. The performance of the new approaches was evaluated in five experiments. Specifically, we evaluated static and adaptive binning in a diabetes mellitus patient, we compared the different decay models in a healthy volunteer and performed a group comparison between diabetes patients and controls. An overview of the visualization capabilities and a comparison of static and adaptive binning is shown for a patient with macular hole. FLIMX’s applicability to fluorescence lifetime imaging microscopy is shown in the ganglion cell layer of a porcine retina sample, obtained by a laser scanning microscope using two-photon excitation.

## Introduction

Fluorescence lifetime imaging ophthalmoscopy (FLIO) is a new technique based on fluorescence lifetime imaging (FLIM) that measures the *in vivo* autofluorescence intensity decays generated by endogenous fluorophores in the ocular fundus. It produces quantitative images based on the lifetimes of the different fluorophores in the fundus and thus extends standard autofluorescence intensity imaging [1, 2]. Each fluorophore possesses a characteristic fluorescence lifetime, which is also influenced by the environment of the molecule (e.g., the surrounding solvent molecules or substances to which it can bind). Thus, fluorescence lifetime measurements offer more information from fluorescence than just the intensity. Furthermore, the fluorescence lifetime is usually independent from the fluorescence intensity.

The goal of FLIO in the human eye is the early detection of eye diseases and other diseases, which might be possible using measurements at the fundus. Another application of FLIO is in basic research, where it can be used, for example, to reveal pathological mechanisms for metabolic diseases [3]. FLIO at the human fundus has the potential to become a valuable diagnostic tool for discovering functional alterations related to eye diseases, such as age-related macular degeneration (AMD), diabetic retinopathy, and glaucoma, before permanent morphological damage occurs. While pathologic changes in the retina are often partially or completely irreversible, metabolic changes are not necessarily permanent and can potentially be reversed. Schweitzer et al. [4] developed FLIO. Recently, fluorescence lifetimes have been determined for subretinal deposits of metabolic byproducts, called drusen, retinal pigment epithelium (RPE) cells, and Bruch’s membrane in histological sections of a human donor eye [5]. Further, changes in fluorescence lifetime parameters have been found in patients with diabetes [6], glaucoma [7] and patients with Alzheimer’s disease [8].

FLIO is based on fluorescence lifetime imaging [9]. fluorescence lifetime imaging techniques are used in microscopy [10] and for *in vivo* tissue characterization and diagnostics [11]. To extract fluorescence lifetime parameters from FLIM data, a least squares based method [12] is often used. Therefore, a number of software solutions are available for FLIM. Enderlein and Erdmann [13] developed the software package FluoFit to fit data with a multi-exponential decay curve. The TIMP software package [14] performs a global analysis (fluorescence lifetime components are assumed to be spatially invariant) utilizing a partitioned variable projection algorithm and includes support for visual interpretation of the results. FLIMFit [15] extends the global analysis to an arbitrary number of fluorescence decay images in predefined spectral bands, supports multi-exponential approximation and visualization of the results. Furthermore, commercial software tools such as SPCImage (Becker & Hickl GmbH, Berlin, Germany) and FluoFit (PicoQuant GmbH, Berlin, Germany) are available.

None of the available software packages can be adapted to the layered structure of the eye, nor can they account for artifacts or correct for the influence of the crystalline lens fluorescence on the retina fluorescence (see sections *Fluorescence of the Eye* and *Approximation of the Fluorescence Lifetime for One Pixel*). In addition, software for performing an in depth analysis of the fluorescence lifetime parameters of a single patient or for performing statistical comparisons of groups of patients is not available. To address these issues, a software package called FLIM eXplorer (FLIMX) has been developed and is introduced in this paper. FLIMX implements known multi-exponential and stretched exponential [16] approaches, as well as new layer-based multi-exponential approaches. To determine the fluorescence lifetime parameters, different stochastic and deterministic minimization algorithms are implemented. A common problem in FLIM and FLIO is a low number of photons in large areas, which can result in incorrect fluorescence lifetimes. FLIMX solves this problem by introducing an adaptive binning approach. FLIMX also offers an approach to correct for the influence of the crystalline lens fluorescence on the approximated fluorescence lifetime of the retina, based on a separate crystalline lens measurement. 2D and 3D visualizations, segmentations, cluster diagrams, and histograms as well as descriptive and advanced statistics facilitate interpretation of the fluorescence lifetime parameters for single patients and groups of patients. To demonstrate the capabilities of the FLIMX software, this software was applied to evaluate a diabetes mellitus patient, a healthy volunteer and a patient with macular hole. It was also used to perform a group comparison between diabetes mellitus patients and controls. Moreover, the FLIMX software was applied to analyze the ganglion cell layer in a porcine retina sample.

## Materials and Methods

### Instrumentation

**Fig 1:**
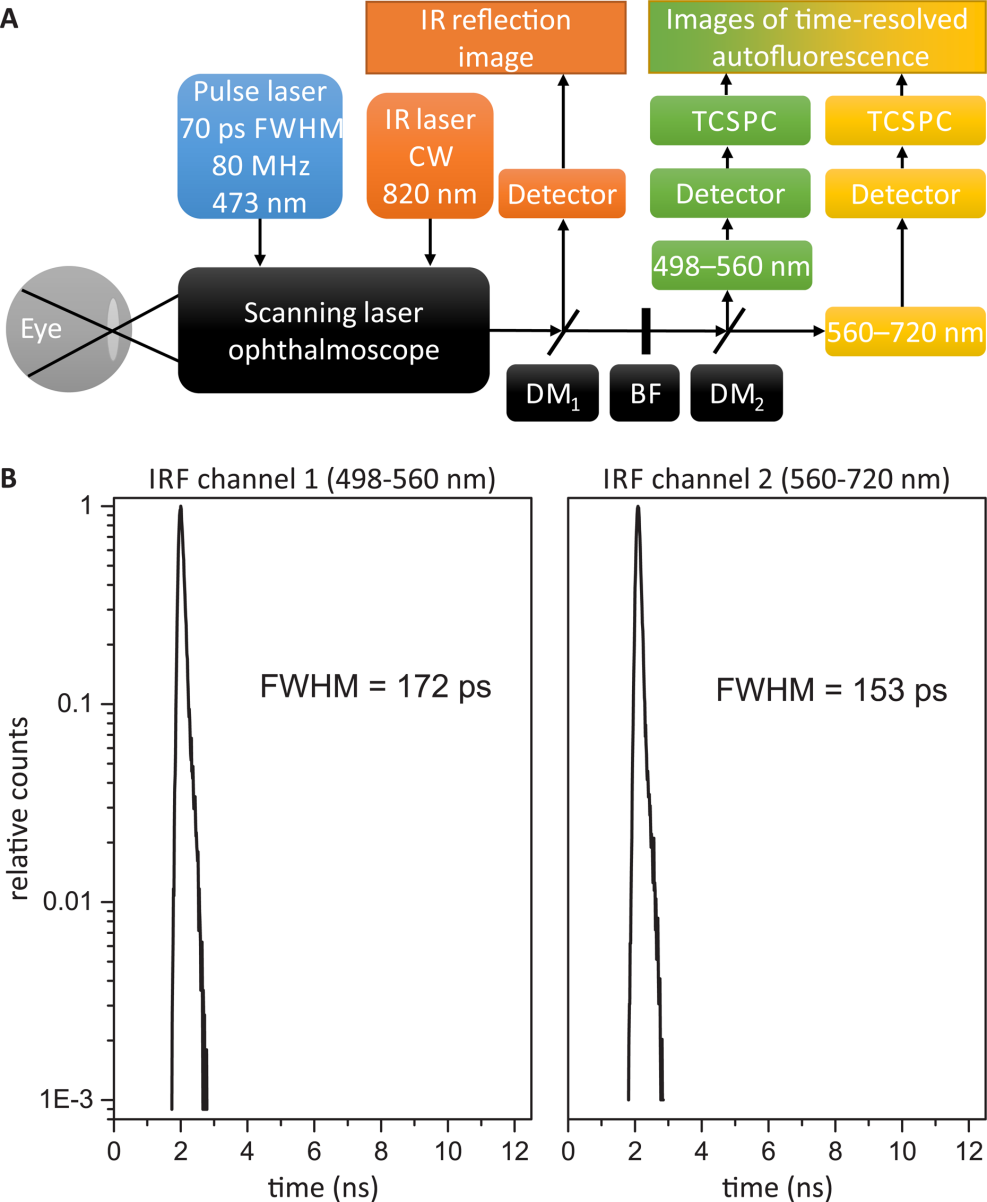
Schematic of the FLIO instrumentation and the instrument response functions. (A) shows a schematic of the FLIO instrumentation. A 473 nm pulse laser is fed into a scanning laser ophthalmoscope to excite the autofluorescence of the eye. The fluorescence emission is transmitted by a multimode fiber to a dichroic mirror (DM), which divides the fluorescence signal into two spectral channels: 498–560 nm and 560–720 nm. Hybrid photomultiplier tube detectors convert the fluorescence photons into electrical pulses, which are processed by a TCSPC device for each detector. A continuous wave (CW) infrared laser (IR) illuminates the fundus for online image registration. Blocking filters (BF) protect the detectors from excitation and infrared light. The FLIO instrument response functions are given in (B).

FLIO instrumentation has been described in detail elsewhere [17, 18]. Thus, only a short description is given here. A schematic of the FLIO instrumentation is shown in Fig 1A.

The basis is a confocal scanning laser ophthalmoscope (cSLO, HRA-2, Heidelberg Engineering GmbH, Heidelberg, Germany). Fundus images (30°, 256x256 pixels) are recorded in high-speed mode at 8.8 frames/s. A pulsed diode laser with a wavelength of 473 nm (BDL-473-SMC, Becker & Hickl GmbH), a pulse width of approximately 70 ps (full width at half maximum) and a repetition rate of 80 MHz is fiber-coupled (single-mode) into the cSLO to excite the autofluorescence. The laser power in the corneal plane is circa 150 μW, well below the exposure limits set by the ANSI standards for durations of up to 8 h [19]. A multimode fiber collects the fluorescence photons and transmits them to filters to block the excitation light. A dichroic mirror (edge-wavelength 560 nm) splits the fluorescence photons into two spectral channels (498–560 nm and 560–720 nm), and there is one detector for each channel (HPM-100-40, Becker & Hickl GmbH). Each detector is connected to a time-correlated single photon-counting (TCSPC) device (SPC-150, Becker & Hickl GmbH). The TCSPC technique [9, 20, 21] generates time-, space- and spectrum-resolved fluorescence decay datasets.

The instrument response function (IRF) was measured using a 25 μM Eosin Y solution which additionally contained 5 M potassium iodide. The Eosin Y solution was prepared by dissolving Eosin Y powder (Sigma-Aldrich Chemie GmbH, Taufkirchen, Germany) in a small volume of dimethyl sulfoxide (DMSO) first. Then water was added to obtain a stock solution of 2 mM which finally was further diluted down to 25 μM using a solution of 5 M potassium iodide. The Eosin Y fluorescence can be excited from 350 to 500 nm and ranges between 450 and 680 nm with a sufficient intensity. Based on the reported Rose Bengal fluorescence lifetime of ca. 16 ps when dissolved in 5 M potassium iodide [22] it is reasonable to assume that Eosin Y, another Fluorescein derivative, shows similar characteristics which could be confirm by in-house measurements. Additionally, no differences in terms of shape and width were found in the IRFs based on the Eosin Y fluorescence in comparison to the IRFs measured using scattered excitation laser light. To measure the Eosin Y fluorescence based IRF, a flat cylindrical quartz cuvette with a detachable window and a volume of 90 μL (124–0.5–40, Hellma GmbH & Co. KG, Müllheim, Germany) was placed slightly tilted in front of the FLIO device. The acquisition time was set to 2–3 minutes which is comparable to typical measurements in volunteers. The IRF images showed no sign of spatial variation. The full width at half maximum of the IRF is 172 ps for channel 1 (498–560 nm) and 153 ps for channel 2 (560–720 nm). The IRFs of both spectral channels are shown in Fig 1B. All IRFs used in this work have similar properties as those in Fig 1B, including experiment 5.

### Fluorescence of the Eye

To measure the fluorescence of the retina using the FLIO instrumentation discussed above, the excitation light has to pass through the lens. A crystalline lens emits a strong autofluorescence when excited at 473 nm [23], which lies mostly in the short wavelength channel (498–560 nm) of the FLIO instrumentation. The excitation pulse generates fluorescence in the crystalline lens before the pulse travels through the vitreous and reaches the retina. The fluorescence of the crystalline lens is isotropic and thus also reaches the retina. The reflectance of the human retina is very low (≤ 2%) as it absorbs most of the incoming light [24]. Further, the confocal properties of the instrument result in a much smaller contribution of the crystalline lens fluorescence reflected from the retina in comparison to the directly detected autofluorescence of the crystalline lens. Because of the large volume and the strong autofluorescence of the crystalline lens, the FLIO instrumentation is not able to suppress the autofluorescence signal of the crystalline lens entirely. The crystalline lens fluorescence will bias the approximated fluorescence lifetime of the retina, especially if the relative contribution of the crystalline lens becomes larger, e.g. in older patients with beginning cataract. The time shift *tc* caused by the distance *d* between the crystalline lens and the retina is defined as:

$$tc=\frac{2\cdot d\cdot n}{c}$$ (1)

where *n* is the refractive index and *c* is the speed of light. For the average Gullstrand Schematic Eye [25], *d* is 22.2 mm (the center of the crystalline lens to the retina) and *n* of the vitreous is 1.3668. From these parameters, the 12.2 ps time resolution of the FLIO instrumentation is sufficient to resolve the resulting *tc* of 202.3 ps. The autofluorescence of the crystalline lens is visible as a shoulder in the rising edge of the fluorescence signal (see section *Approximation of the Fluorescence Lifetime for One Pixel*).

### Binning

In FLIO, the spatially and time resolved TCSPC datasets are typically collected with an average number of 1000 photons per pixel in the macular region. This number is a compromise between signal-to-noise ratio (SNR) and acquisition time. If the number of photons is too low, e.g., for a multi-exponential approximation using three exponential functions, the photons of neighboring pixels are combined. This process sums up the decay signals for a square shaped window around each pixel and is called static binning. The edge length *l* of the window is:

l=2⋅f+1 (2)

where *f* is the binning factor. Static binning is effectively a moving average filter in the spatial dimensions. Often, a binning factor of one is used for multi-exponential approximation with two exponential functions, and a binning factor of two is used for multi-exponential approximation with three exponential functions.

The fluorescence intensity of the human retina is not homogeneously distributed. The optic disc is generally weakly fluorescent. High concentrations of macular pigment in the fovea centralis absorb more of the excitation light than is absorbed in the outer regions, and vessels block the excitation light, prohibiting retinal fluorescence from underlying tissue. Another important factor that can contribute to the inhomogeneous distribution of fluorescence is suboptimal alignment of the instrument to the patient. In darker regions with low fluorescence signals, static binning with a fixed binning factor may not collect enough photons for the chosen approximation model, resulting in inaccurate estimates of the fluorescence lifetimes. Choosing a higher static binning factor would collect a sufficient number of photons in darker regions, but at an unnecessary cost of spatial resolution in brighter regions. Furthermore, the number of pixels involved in static binning increases with the binning factor by a power of two, making high binning factors unfavorable.

**Fig 2:**
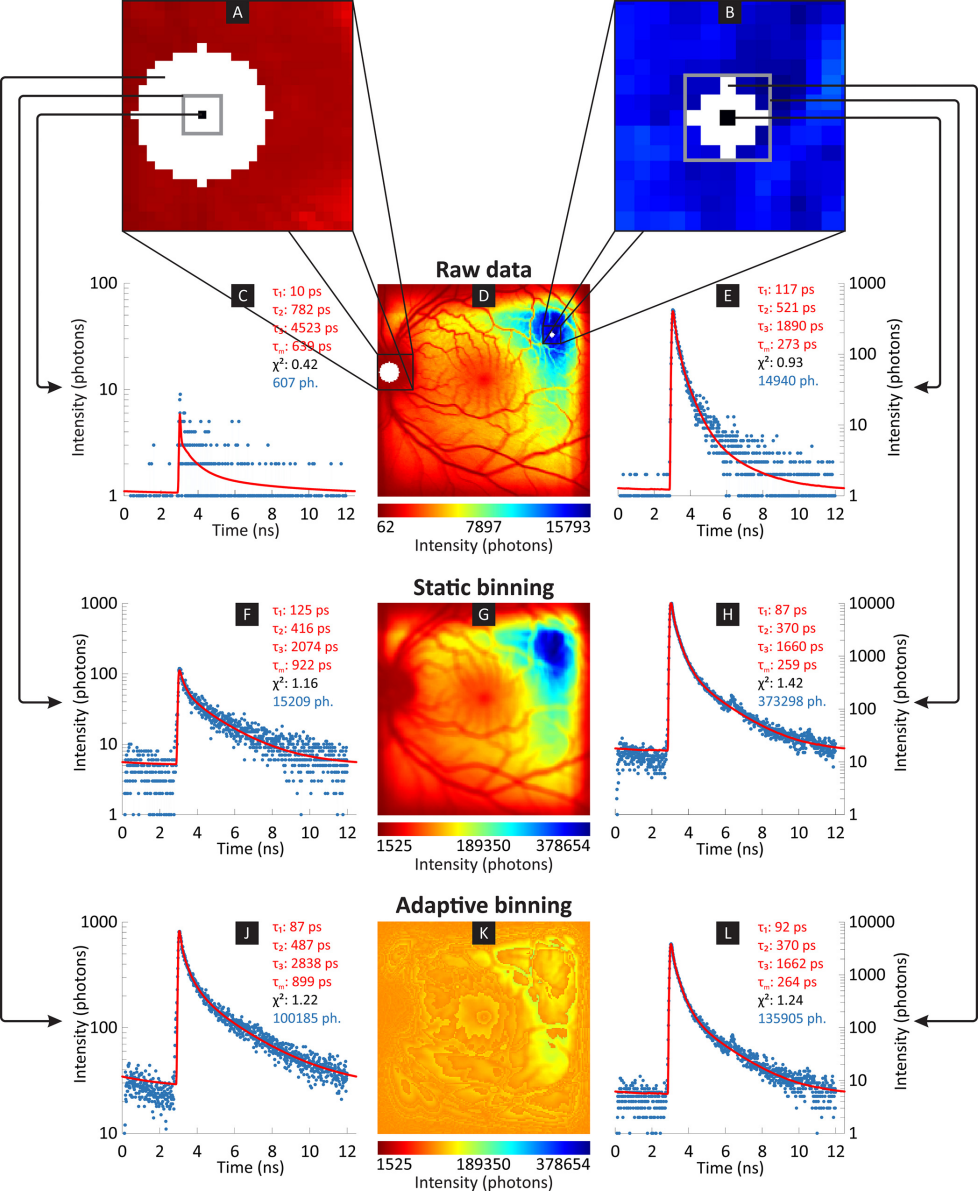
Comparison of static and adaptive binning. TCSPC data obtained from a 39 year old diabetes patient is evaluated. The top row of the photon histograms displays the raw data (C, E), the middle row shows the photon histograms after static binning (F, H) and the bottom row is after adaptive binning (J, L). In the magnified insets (A, B), static binning uses all of the pixels inside the gray box, while adaptive binning uses the white pixels (note the different sizes of the insets). The small black square indicates the seed pixel for the binning. Static binning uses more neighboring pixels for each bright pixel than adaptive binning (right inset, B), while adaptive binning uses more neighboring pixels for each dark pixel than static binning (left inset, A). The left column (C, F, J) shows photon histograms for one pixel (black square in A) in a dark region of the intensity image, while the right column (E, H, L) shows photon histograms for one pixel in a bright region. The middle column displays the fluorescence intensity image (170 x 170 pixels, 59 x59 μm^2^/pixel) of the raw data (D), after static binning (G) and after adaptive binning (K). The greater loss of spatial contrast with adaptive binning yields a more balanced SNR in the photon histograms of the underlying binned pixels. The data in the photon histograms were analyzed using a multi-exponential model (Eq 3) with three exponential functions in combination with incomplete decay (Eq 9). The resulting curve (red) and the corresponding fluorescence lifetimes, the average fluorescence lifetime, the figure of merit χ^2^ (Eqs 10 and 11) as well as the total number of photons are shown next to the photon histogram.

We propose a new adaptive binning approach that overcomes the disadvantages of static binning by using a circular window centered on each pixel. The radius of the circle is iteratively increased until a threshold with a predefined minimum number of photons is reached. Thus, adaptive binning ensures that the required number of photons per pixel is collected at the highest possible spatial resolution. Fig 2 shows a comparison of static and adaptive binning for measuring the fluorescence intensity from a patient with diabetes mellitus. Specifically, it shows the effects of static binning with a binning factor of two (Fig 2G) and adaptive binning with a threshold of 100,000 photons (Fig 2K) for the same patient using identical color scaling. It can be seen that adaptive binning results in a much more homogeneous intensity distribution because each pixel possesses at least 100,000 photons. Furthermore, the intensity drop off at the borders of the image is no longer present. Ideally, the fluorescence intensity distribution after adaptive binning would be completely homogeneous. The loss of global structural information in the fluorescence intensity is irrelevant because the fluorescence lifetime is usually independent of the fluorescence intensity, as stated above. Consequently, adaptive binning has no relevant effect on the fluorescence lifetime distributions. The data in the photon histograms were analyzed using a multi-exponential model (Eq 3) with three exponential functions in combination with incomplete decay (Eq 9). The resulting fluorescence lifetimes as well as the figure of merit χ^2^ (Eqs 10 and 11) are shown next to the photon histogram. The fluorescence lifetimes for the bright pixel differ between static (Fig 2H) and adaptive binning (Fig 2L) for less than 6%. In case of a dark pixel, the fluorescence lifetimes differ between static (Fig 2F) and adaptive binning (Fig 2J) for 17%–44%.

### Modelling of TCSPC Data

Different approaches can be used to describe the decay of the time-resolved fluorescence data [9, 26]. This work concerns the multi-exponential approach, the stretched exponential approach, the spectral global analysis approach, the layer-based approach and the lens-corrected approach, as well as modelling of incomplete decays, all of which are implemented in FLIMX.

The multi-exponential approach describes the data with a sum of the exponential decay curves:

$$I(\text{t})=IRF*\sum_{i}\alpha_{i}\cdot e^{-\frac{t}{\tau_{i}}}+b$$ (3)

where *I* is the time-dependent fluorescence intensity, *IRF* is the instrument response function, *α* is the amplitude, *τ* is the fluorescence lifetime, *i* is the index of the exponential, the asterisk denotes a convolution integral, and *b* is the background, e.g., from thermal noise of the detector or background light. This method seems beneficial for measuring the time-resolved autofluorescence of the human eye in different spectral channels to determine differences between patients suffering from early AMD and healthy controls [27].

It is not feasible to use more than three exponential functions in Eq 3 because of the extremely high number of photons required [28]. According to our experience, between 100.000 and 400.000 photons are required for a reliable modelling with three exponential functions. In FLIO measurements, usually 1.000–10.000 photons/pixel are acquired. Thus, the required number of photons/pixel has to be achieved by binning adjacent pixels using an approach described above. The number of fluorophores in the human eye is larger than three, because there are a number of known fluorophores, including lipofuscin / N-retinyliden-N-retinylethanolamin (A2E), retinal, advanced glycation end products, collagens, flavines and possibly nicotinamide adenine dinucleotide in its reduced form (NADH). Thus, alternative modeling approaches such as the stretched exponential have been introduced [16]. A stretched exponential is able to model the distribution of fluorescence lifetimes by introducing the stretching exponent *β*:

$$I(\text{t})=IRF*\left(\alpha \cdot e^{-{\left(\frac{t}{\tau }\right)}^{\beta }}\right)+b$$ (4)

Some fluorophores are present in one of the two spectral channels, while others might be present in both spectral channels. Because the fluorescence lifetime is independent of a fluorophore’s spectral properties, the fluorescence lifetime is identical in both spectral channels. To exploit this fact, a spectral global analysis for certain exponential functions can be performed. This method can improve the fluorescence lifetime approximation because more photons are used in the analysis.

$$I(\text{t})=\sum_{\lambda }\left(IRF_{\lambda }*\sum_{i}\alpha_{i,\lambda }\cdot e^{-\frac{t}{\tau_{i,\lambda }}}+b_{\lambda }\right)$$ (5)

where *λ* is the identifier of the spectral channel, and at least a single fluorescence lifetime is identical in both spectral channels, *τ*_*i*,*λ1*_ = *τ*_*i*,*λ2*_.

The layered structure of the eye causes fluorescence photons from structures that are farther away from the laser scanner ophthalmoscope to arrive later because of the additional travel time required for the excitation light and the fluorescence photons. To account for distance, Schweitzer et al. [29] enhanced the multi-exponential model by a time shift parameter *tc*, which is called the layer-based approach here:

$$I(\text{t})=IRF*\sum_{i}\alpha_{i}\cdot e^{-\frac{t-tc_{i}}{\tau_{i}}}+b$$ (6)

Stretched exponentials (Eq 4) and the layer-based approach (Eq 6) can be combined for different layers of stretched exponentials:

$$I(\text{t})=IRF*\sum_{i}\alpha_{i}\cdot e^{-{\left(\frac{t-tc_{i}}{\tau_{i}}\right)}^{\beta }}+b$$ (7)

Time-resolved fluorescence measurements of the crystalline lens show that a single exponential function is not sufficient to describe its decay behavior [30]. Instead of approximating the fluorescence lifetimes in a mixture of the crystalline lens fluorescence and the retinal fluorescence, the fluorescence decay of the crystalline lens can be directly embedded into the model. Therefore, the fluorescence decay of the crystalline lens *I*_*lens*_ has to be measured separately. As the distance between FLIO instrument and eye is different for the measurement of the crystalline lens for technical reasons, its fluorescence signal has to be shifted in time using *tc*_*lens*_. The contribution of the crystalline lens fluorescence is modeled by *α*_*lens*_. This approach is called the lens-corrected approach here:

$$I(\text{t})=IRF*\sum_{i}\alpha_{i}\cdot e^{-{\left(\frac{t-tc_{i}}{\tau_{i}}\right)}^{\beta }}+\alpha_{lens}\cdot I_{lens}(\text{t}-{\text{tc}}_{lens})+b$$ (8)

Often, the fluorescence signal does not decay sufficiently in the time period *t*_*R*_ between two laser pulses, which is the reciprocal of the pulse repetition rate. This so-called incomplete decay can be taken into account using an analytic approach [31] which does not correct the pre-excitation interval. Thus, a numerical approach is used here which also corrects the pre-excitation interval:

$$I(\text{t})=I_{C}(t)+\sum_{j}I_{C}(t+j\cdot t_{R})$$ (9)

where *I*_*C*_ is the fluorescence intensity calculated according to Eqs 3–8 and *j* is the number of time periods to consider. The choice of *j* depends on the fluorescence lifetimes occurring in the sample, as the exponential components have to be decayed sufficiently in the *j∙t*_*R*_ time-frame. In FLIO with a *t*_*R*_ = 12.5 ns, a *j = 1* is usually enough for the expected fluorescence lifetimes of up to circa 5 ns.

### Approximation of the Fluorescence Lifetime for One Pixel

**Fig 3:**
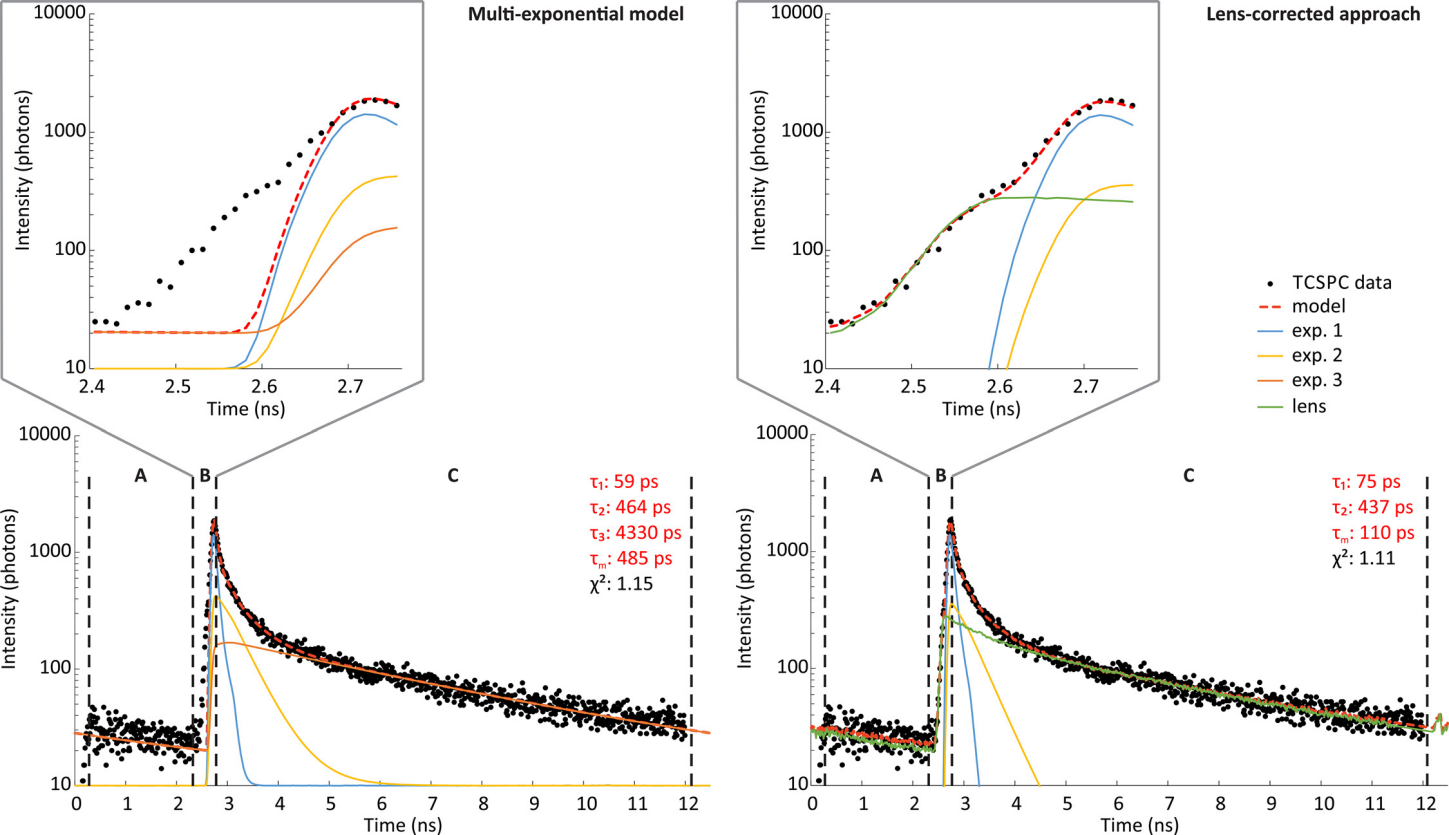
Example of TCSPC data approximated by the multi-exponential model and the lens-corrected approach. TCSPC data obtained from a healthy volunteer is evaluated using adaptive binning (black) and approximated by the multi-exponential approach (left) using three exponential functions and the lens-corrected approach (right) using two exponential function and a separate measurement of the crystalline lens. The data are divided into three intervals: the pre-excitation interval (A), the fluorescence rising edge (B) and the fluorescence decay (C). The measured data and the multi-exponential model diverge due to the fluorescence of the crystalline lens in interval (B), which is magnified in the inset. The lens-corrected approach utilizes a scaled and shifted curve of a separate measurement of the crystalline lens to correct for the influence of the crystalline lens fluorescence in interval (B). For better visibility, the fluorescence intensity is plotted on a logarithmic scale. The fluorescence lifetimes of the exponential components (τ_1_, τ_2_, τ_3_), the average fluorescence lifetime τ_m_ as well figure of merit χ^2^ (Eqs 10 and 11) are shown next to the photon histogram.

An example of a time-resolved fluorescence signal from a single fundus-pixel from a healthy volunteer, approximated using the multi-exponential model (Eq 3) and the lens-corrected approach (Eq 8), is shown in Fig 3. There are three main intervals in the fluorescence signal: the pre-excitation interval, the fluorescence rising edge and the fluorescence decay. Usually in FLIM, the shape of the excitation pulse, the detector response function, dispersion in the optical pathway as well as relaxation processes directly after excitation and immediately before fluorescence emission determine the rising edge. In FLIO, the autofluorescence of the crystalline lens also affects the rising edge, as discussed above. The autofluorescence of the crystalline lens is visible as a shoulder in the rising edge in Fig 3 (inset).

To approximate the fluorescence lifetime of the retina using Eqs 3–5, only the decay of the fluorescence signal is important. For Eqs 6–8, the rising edge of the fluorescence signal must be taken into account, in addition to the decay.

If the fluorescence lifetimes of a sample are sufficiently short, the pre-excitation interval can be used to calculate the background *b*. If incomplete decays are considered (Eq 9), it is often useful to include the pre-excitation interval data as well.

To approximate the fluorescence lifetime parameters, a global optimum characterized by the smallest possible figure of merit must be found. To quantify the figure of merit, the *χ* ^*2*^ error in its reduced form *χ*_*r*_^*2*^ is used:

$$\chi_{r}^{2}=\frac{1}{m-p}\sum_{j=1}^{m}\frac{{\left(I_{M}(t_{j})-I_{C}(t_{j})\right)}^{2}}{w(t_{j})}$$ (10)

where *m* is the number of time channels of the photon histogram, *w(t*_*j*_ *)* is the weighting in time channel *j*, *I*_*M*_ *(t*_*j*_ *)* is the number of measured photons, *I*_*c*_ *(t*_*j*_ *)* is the number of calculated photons using one of the models discussed above and *p* is the number of free parameters in the model. The ideal weighting is *w(t*_*j*_ *) = ϭ*_*j*_^*2*^, where *ϭ*_*j*_ is the standard deviation of the number of measured photons, which is the square root of the expected value for data with a Poisson distribution. To approximate the weighting, the Neyman approach [32] is used:

$$w\left(t_{j}\right){=I}_{M}\left(t_{j}\right)$$ (11)

In addition, other weighting approaches such as Pearson [32], fitted weighting [33] and Warren [15] have been implemented in FLIMX as the choice of the weighting is a critical factor [33].

To find the global optimum of *χ*_*r*_^*2*^, a minimization scheme is required. The summation of the exponentials is a linear operation. Thus, a linear minimization algorithm can determine the amplitudes as well as the background. The remaining parameters must be determined by a non-linear minimization algorithm. Generally, there are two types of non-linear minimization algorithms: stochastic approaches, such as the evolution strategies [34] and the particle swarm method [35], and deterministic approaches, such as the Levenberg-Marquardt method [36] and the Nelder-Mead simplex method [37]. Deterministic approaches are dependent on their initial solution and often cannot determine the global optimum. Stochastic approaches are based on random variation and can find the global optimum, but at the cost of a higher computational effort.

The following minimization algorithms are implemented in FLIMX: differential evolution [38], a particle swarm variant [39], and a modified version of the Nelder-Mead simplex method [37]. Any of them can be applied to a pixel-wise fluorescence lifetime approximation. The stochastic minimization algorithms deliver robust results but require 10 to 100 times more computation time. Thus, a stochastic minimization algorithm can be used, for example, to estimate an initial solution from a single fluorescence decay profile that is based on an integral of the fluorescence decay profiles across the image. In a second step, a pixel-wise approximation utilizes a deterministic algorithm, with this initial solution as starting point. Using this procedure and Eq 3 to compute the fluorescence lifetimes of an image with 256 x 256 pixels, 33 / 44 / 100 minutes were required using one / two / three exponential functions (dataset from experiment 3, 4.00 GHz Intel Core i7 4790K quad-core processor with 16 GB of memory).

All minimization algorithms implement parameter constraints to allow only positive values for the fluorescence amplitudes, the fluorescence lifetimes and the background. The stretching exponent is restricted to values between zero and one. Custom constraints can also be defined to exploit a priori knowledge, e.g., if a certain fluorescence lifetime is expected to occur in the sample.

### Handling of Artifacts in the Fluorescence Lifetime Signal

**Fig 4:**
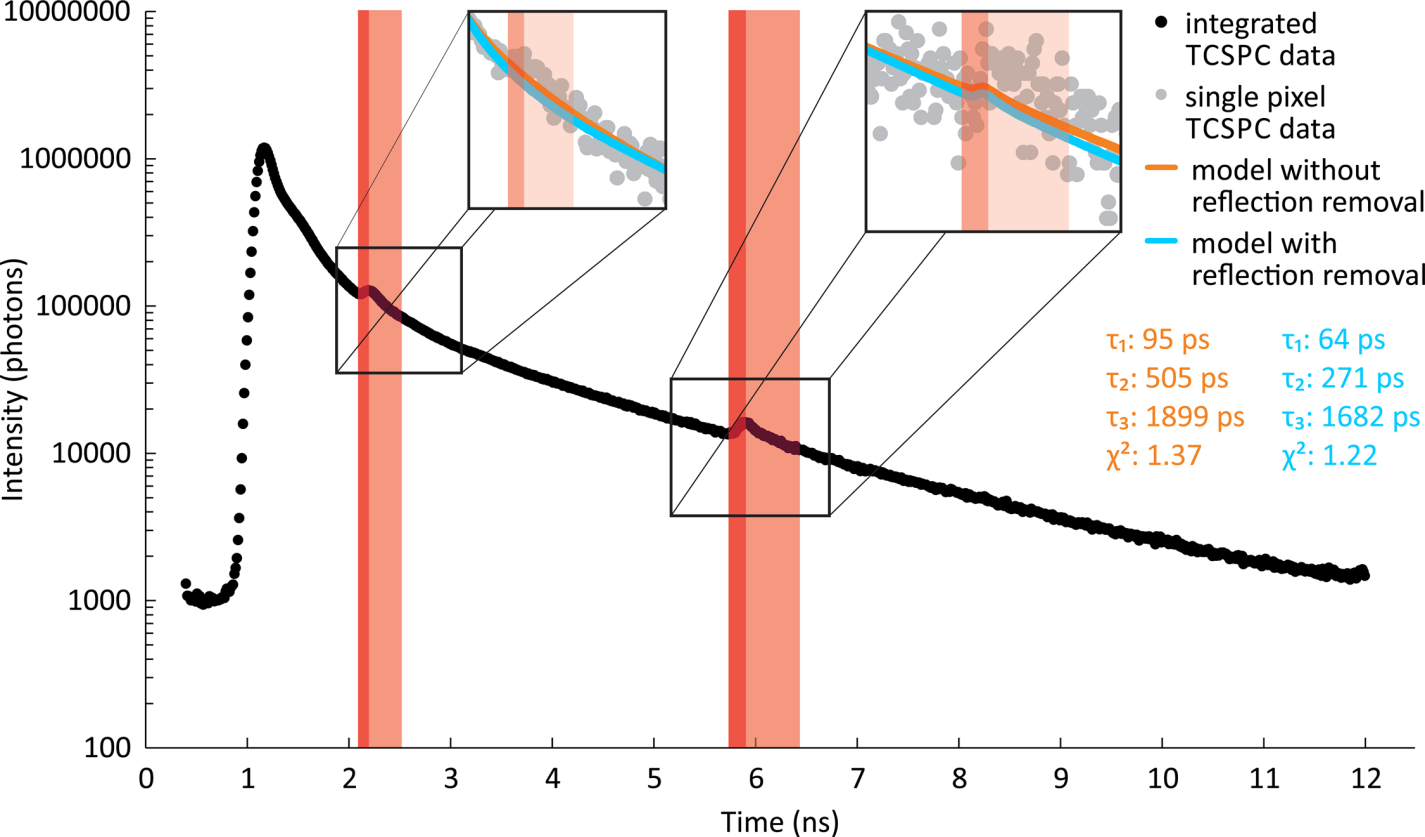
Reflection artifacts in TCSPC data after binning all of the pixels of an image. TCSPC data after binning all of the pixels of an image from a healthy volunteer (black). The automatically detected artifacts caused by reflections in the optical pathway are divided into the rise of the artifact (dark red) and the decay of the artifact (light red). The latter is approximated as three times the width of the rise. For better visibility, the fluorescence intensity is plotted on a logarithmic scale. The insets show the magnified segment of the TCSPC data taken from a single pixel (gray). The results of a multi-exponential analysis (Eq 3) using three exponential functions are shown as orange (without removal of the reflection artifacts) and blue curves (with removal of the reflection artifacts).

Reflections in the optical pathway cause artifacts in the photon histogram of the fluorescence decay. The fluorescence signal decreases over time, starting at the excitation. A reflection of the fluorescence photons at two or more reflective surfaces/interfaces, e.g., at the surface of a detector or an optical filter, causes a sudden increase in the fluorescence signal at some point in the decay because the reflected photons travel an additional distance and, therefore, arrive at a later point in time. An example of a reflection artifact is shown in Fig 4. Usually such artifacts are eliminated by avoiding surfaces perpendicular to the optical pathway and by applying antireflection coating to surfaces and fiber ends. However, some residual reflection artifacts often remain. Thus, an algorithm to detect and remove reflection artifacts was developed. First, because the positions of the reflection artifacts in the time dimension are invariant for all pixels, all of the pixels of the image are binned into a single fluorescence decay, resulting in the best possible SNR. The algorithm then searches the gradient of the fluorescence decay for sections with a rising gradient to find the starting time of the artifact. The end time is approximated by adding three times the interval from the starting time to the peak time of the reflection to the starting time. This procedure was determined empirically using FLIO data. For data with longer fluorescence decays than usually occurring in FLIO the estimation of the end time would have to be adjusted. In Fig 4, the detected reflection artifacts are marked in red, with the sections of the rising gradient colored in dark red and the estimated decay of the artifact in light red. In contrast to Fig 3, there is much less noise in the fluorescence decay due to the binning of all of the pixels in the image. The detected reflection artifacts are removed by excluding the time intervals of the artifacts from all of the computations for the figures of merit for each pixel.

### Improving Approximation by Exploiting Spatial Information

A number of factors affect the approximation of the fluorescence lifetime parameters when utilizing the model function and minimization algorithm discussed above. For example, noise due to a low number of measured photons can lead to unstable results in the approximation. An ill-posed model function, e.g., due to a higher number of excited fluorescent substances than employed exponential functions, can increase the ill-posedness of the problem. Furthermore, the minimization algorithm may be stuck at a local minimum and, thus, will not be able to find the global minimum. Spatial a priori information can be used to improve the approximation. In FLIO with a spatial resolution of circa 34 x 34 μm^2^ per pixel, the fluorescence lifetime properties of a biological tissue are expected to change only moderately from pixel to pixel. Thus, the fluorescence lifetime parameters for a certain pixel should also provide reasonable initial approximation for its surrounding pixels. To model a pixel, a figure of merit may also be computed for the adjacent pixels to achieve a spatially smoother fluorescence lifetime approximation. The resulting $\chi_{r,N}^{2}$ is defined as the combination of the figure of merit according to Eq 10 for the central pixel $\chi_{r,\text{CP}}^{2}$ and the k’th adjacent pixels $\chi_{r,\text{APk}}^{2}$ fitted using the parameters from central pixel:

$$\chi_{r,N}^{2}={\left(\chi_{r,\text{CP}}^{2}\right)}^{2}+\left(\frac{1}{n}\sum_{k}^{n}\chi_{r,\text{APk}}^{2}\right)$$ (12)

where *n* is the number of adjacent pixels.

### Iterative Algorithm for the Treatment of Outliers

**Fig 5:**
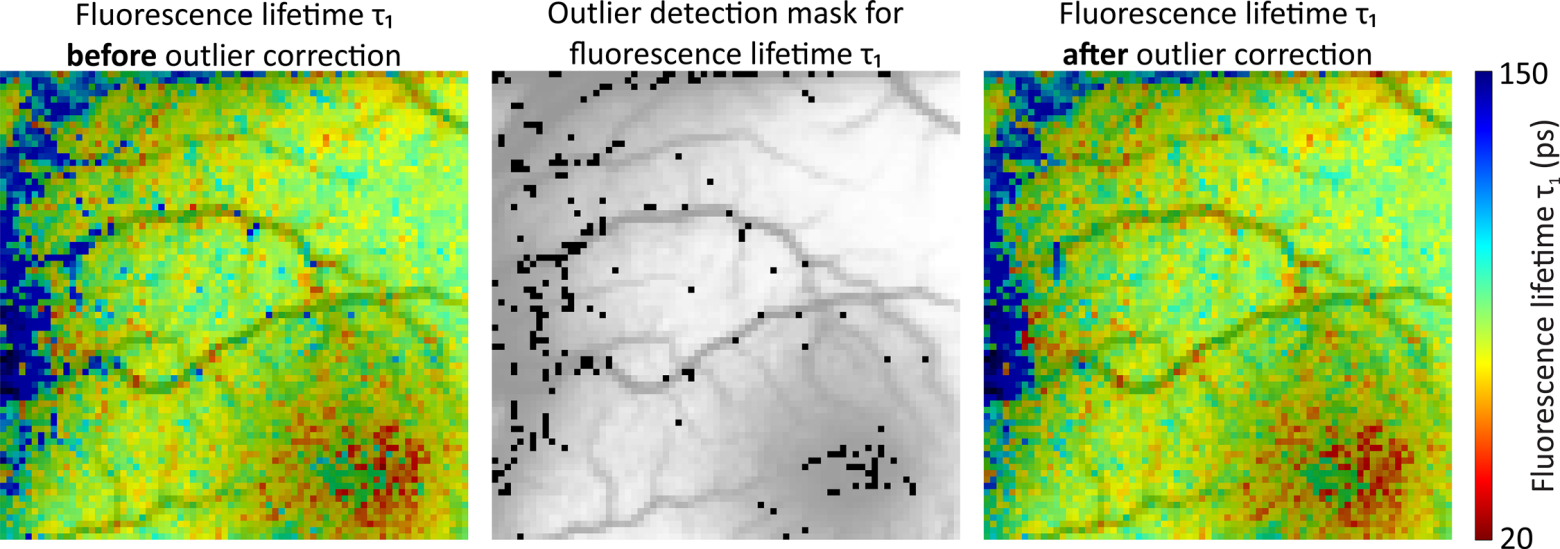
Example of the iterative algorithm for the treatment of outliers. Comparison of a 75 x 75 pixels section (59 x59 μm^2^/pixel) of fluorescence lifetime τ_1_ from the left eye of a healthy volunteer before (left) and after (right) correction for outliers. The color scaling of both fluorescence lifetime plots is identical. The 259 detected outliers are colored black in the middle subplot, of which 178 could be improved to generate the corrected image. To provide better orientation, a gray scale image of the fluorescence intensity has been added as an overlay to all three subplots. The macula is in the lower right corner of the image, where the shortest fluorescence lifetimes (red) occur.

Because outliers in the fluorescence lifetime images may be present, a separate treatment of these outliers might be needed to improve the fluorescence lifetime approximation. To search for outliers after the fluorescence lifetime is approximated for all of the pixels of an image, a sliding window of a user-defined size is moved over the image. A window size of 7 x 7 pixels showed good results in practical tests. The window is moved through the images of the fluorescence lifetime parameters and the figure of merit. In each window, the central pixel is compared to the median of all of the pixels inside the window. If the relative difference between a pixel and the median is above a user-defined threshold, the approximation of this pixel is repeated using the fluorescence lifetime parameters from the pixel with the best figure of merit inside the window as initial values. The result of the repeated fluorescence lifetime approximation is accepted only if its figure of merit is better than the original approximation. The applied threshold depends on the application and the expected changes in the parameter space. Thresholds of circa 30 percent demonstrated good results in our applications. Fig 5 illustrates the outlier correction procedure. In the example, 259 outliers were detected and of those, 178 could be improved in one iteration of the algorithm described above. Multiple iterations of the outlier treatment could improve even more outliers.

### Average Fluorescence Lifetime and Spatial Filtering

For some applications, the average fluorescence lifetime *τ*_*m*_ is a good overview parameter. It can be derived from the fluorescence amplitudes and lifetimes:

$$\tau_{m}=\frac{\sum_{i}\alpha_{i}\cdot \tau_{i}}{\sum_{i}\alpha_{i}}$$ (13)

To reduce noise in the fluorescence parameter space, spatial mean- or median-filtering can be applied to the obtained images. A reasonable size for the filter kernel is either 3 x 3 or 5 x 5 pixels, depending on the amount of noise in the data. The statistics discussed in the section below are calculated using the filtered data.

### Regions of Interest

Although visual analysis is important for a general judgment of the image quality, quantitative analysis is required for the detection of pathologic alterations. Dysli et al. [40] proposed to use the ETDRS grid introduced by the Age-Related Eye Disease Study group [41] to define standardized regions of interest (ROI). FLIMX implements the ETDRS grid (see the results sections of experiments 1 and 2 for exemplary applications of the ETDRS grid). Three concentric circles in the center of the macula and four radial lines at 45°, 135°, 225° and 315° compose the grid. The radii of the circles are related to the diameter of the optic disk of an average eye, which is 1500 μm. Specifically, the radius of the inner circle corresponds to 1/3 of the optic disk diameter, the radius of the middle circle is equal to 1 optic disk diameter, and the radius of the outer circle is equal to 2 optic disk diameters. Hence, these radii of the inner, middle, and outer circles are 500, 1500, and 3000 μm, respectively. Based on this grid, nine subfields are defined: central, inner superior, inner inferior, inner nasal, inner temporal, outer superior, outer inferior, outer nasal, and outer temporal. Users can also define a custom rectangular ROI. For each subfield of the ETDRS grid or a custom ROI, descriptive statistics including the mean, median, mode, standard deviation, variance and confidence intervals are used to quantitatively compare groups of patients. Histograms with user-defined class widths are also computed for the ETDRS grid subfields and custom ROIs.

### Group Comparison

The Holm-Bonferroni method [42] is used for group comparisons. First, a histogram for a certain fluorescence lifetime parameter, e.g., τ_1_, is computed for each volunteer. Then, for each class in the histogram, a two-sided Wilcoxon rank sum test [43] is performed on the patient data against the controls. This approach tests the null hypothesis that the data from the patients and controls are samples from continuous distributions with equal medians against the alternative that they are not, at a certain significance level. The p-values from the test are compared to a threshold *th*:

$$th=\frac{s}{nC}$$ (14)

where *s* is the significance level and *nC* is the number of histogram classes for the fluorescence lifetime parameter. Histogram classes with p-values smaller than the threshold possess a significant difference in medians between the patients and controls. If such a significant difference is found for a histogram class, it can be used as a classifier. Subsequently, its specific cut off point as well as its sensitivity and specificity are determined. The Holm-Bonferroni method can then be applied to the full image or to a region of interest, such as a subfield of the ETDRS grid. In the latter case, only pixels inside the ROI are used for the histogram and for the statistics of the Holm-Bonferroni method. The Holm-Bonferroni method is applied to an exemplary group comparison in experiment 4.

### Implementation

FLIMX was implemented in MATLAB (The MathWorks, Inc., Natick, Massachusetts, USA), mostly in an object-oriented programming style, as shown in S1 Fig. FLIMX includes a patient database and has the ability to group patients in studies. For each patient, single measurements and, if calculated, the corresponding results of the fluorescence lifetime approximation, both divided into spectral channels, are stored in the database. The backbone of FLIMX is a tree-like data structure, which stores studies and patients and handles disk access, as depicted in S2 Fig. The measured data are imported into FLIMX only once and are then saved internally. Auxiliary information, such as age, gender, disease state, can be stored for each patient and can later be used to define subgroups in a study. Patients can be copied or moved between studies. The parameters for binning, data modelling, minimization, constraints and computation are also saved by FLIMX, making it possible to trace the study settings from calculated results. Computational routines are separated into modules, which implement the methods described in this work. FLIMX’s fluorescence lifetime approximation requires the user to supply an IRF. The IRFs used in this work and the measurement procedure are described in the instrumentation section. Graphical user interfaces are available for all aspects of FLIMX, such as changing settings, altering the data structure, visualizing fluorescence lifetime parameters and statistics, as well as for access to export abilities.

**Fig 6:**
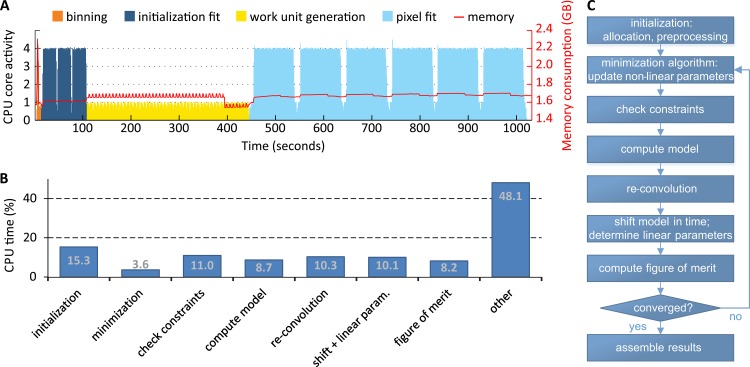
Profile of the CPU and memory requirements for FLIMX. (A) shows the fractional CPU core activity for a quad core processor and MATLAB’s total memory consumption (red) during a distributed fluorescence lifetime approximation of a measurement. (B) shows a breakdown of the CPU time spent on the different stages of the algorithm (C).

A tool for generating synthetic, time-resolved fluorescence data is also included in the software package. It can be used for the simulation processes, e.g., for the construction of the fluorescence decays based on the fluorescence lifetimes of known fluorophores. In addition, a batch job manager is included, which allows for the assembly and batch processing of approximation jobs. The software can handle an arbitrary number of studies and patients with different parameter sets. The computation of the fluorescence lifetime parameters can be very time consuming, depending on the selected minimization algorithm. Thus, FLIMX is able to run the computation on different pixels in parallel, utilizing multiple CPU cores. A custom built backend allows for the user-transparent distribution of work units across multiple compute servers. Therefore, a slave process is started on each server, which scans a network folder for the work unit files generated by the FLIMX software. Each work unit can contain several pixels, which are computed in parallel by the slave process using up to 12 CPU cores (the current maximum number of parallel processes per MATLAB session). The results of the computation are saved in the shared folder, and FLIMX uses the results to assemble the images of the fluorescence lifetime parameters. File access is synchronized using semaphores. The CPU core activity and memory requirements for a typical distributed fluorescence lifetime approximation using the lens-corrected approach on a 256 x 256 pixel dataset, obtained from a healthy volunteer, are given in Fig 6A. The critical code paths of the fluorescence lifetime approximation have been optimized for processing speed. An overview of the CPU time spent on the different stages of the fluorescence lifetime approximation is shown in Fig 6B. A large amount of the computation time is not spent in specific stage of the algorithm, but on other things such as memory allocation, consistency checks, object creation, function calls and general MATLAB overhead. The benchmarks were performed on a 2.93 GHz Intel Core i7 940 quad-core processor with 12 GB of memory.

To analyze the approximation results, fluorescence lifetime parameters can be visualized in two- and three-dimensions. The three-dimensional view can be freely rotated. Arithmetic operations between different fluorescence lifetime parameters and different spectral channels enable an in-depth analysis of the approximation results.

### Example Data

#### Preface

All research procedures were performed according to the Declaration of Helsinki. Approval for the study was obtained from the ethics committee of the Jena University Hospital. Written informed consent was obtained from each volunteer prior to participation in the study.

The following methods were used for all experiments. For Eqs 3–5, only the decay of the fluorescence signal was used for the fluorescence lifetime approximation. In case of Eqs 6–8, the whole fluorescence signal, including pre-excitation interval and rising edge, was used. The figure of merit (Eq 10) was computed using Neyman weighting (Eq 11). As discussed above, an initial solution for the fluorescence lifetimes of the whole image was computed using the differential evolution algorithm. For the pixel-wise approximation of the fluorescence lifetimes, the Nelder-Mead simplex method was applied with the initial solution as starting point. Incomplete decay (Eq 9) and the treatment of outliers were applied.

#### Experiment 1: patient with diabetes mellitus

FLIO data were measured in the left eye of a 39-year-old male patient with diabetes mellitus type 1 without diabetic retinopathy and a crystalline lens. The motivation of this experiment is to demonstrate the abilities of our adaptive binning approach in comparison to static binning. Therefore, static binning with a binning factor of two and adaptive binning with a threshold of 100,000 photons per pixel were applied to the FLIO data. The fluorescence lifetime parameters were determined using the lens-corrected approach (Eq 8), with two exponential functions, ß set to 1 and a separate crystalline lens measurement.

#### Experiment 2: healthy volunteer

FLIO data were measured in the left eye of a 59-year-old male healthy volunteer with a hemorrhage located in the superior temporal region. The patient had a crystalline lens. The adaptive binning approach with a threshold of 100,000 photons per pixel was utilized. To determine the fluorescence lifetime parameters, the following approaches were applied separately:

- a multi-exponential model using three exponential functions (Eq 3)
- the spectral global analysis approach (Eq 5) using three exponential functions, with the short component fixed for both spectral channels
- the layer-based approach (Eq 6) using three exponential functions, with the long component able to shift on the time axis
- the layer-based approach in combination with two stretched exponentials (Eq 7), with one of them able to shift on the time axis
- the lens-corrected approach (Eq 8), with three exponential functions, ß set to 1 and a separate crystalline lens measurement

The results were compared using descriptive statistics.

#### Experiment 3: patient with macular hole

FLIO data were measured in the left eye of a 68-year-old female patient with a macular hole without overlying operculum. A macular hole is a structural defect in the sensory retina at the site of the highest visual acuity. The patient had a crystalline lens. The FLIO data, which were binned by static binning with a binning factor of two, were analyzed using a multi-exponential model (Eq 3) with three exponential functions. In addition, the FLIO data were binned by the adaptive binning approach with a threshold of 100,000 photons per pixel and analyzed using the lens-corrected approach (Eq 8), with two exponential functions, ß set to 1 and a separate crystalline lens measurement.

#### Experiment 4: diabetes patients and healthy controls

The motivation of this experiment is to show the ability of our analysis chain to provide group comparison for groups of patients or volunteers. This is important because the high inter-individual variability might not allow for subject specific discrimination for all FLIO applications. We exemplify this group comparison on 20 diabetes mellitus patients without diabetic retinopathy, aged 63.9 ± 8.2 years, and 21 controls, aged 59.1 ± 11.3 years. All volunteers had a crystalline lens. The FLIO data, which were binned by static binning with a binning factor of two, were analyzed using a multi-exponential model (Eq 3) with three exponential functions. A 71x101 pixels region in the superior temporal location of the fundus, which included the macula, was manually segmented in all of the patients and controls. Based on this region, significant differences between patients and controls were determined using the Holm-Bonferroni method, described in the group comparison section above. The following histogram class widths were used: amplitudes of 1%; fluorescence lifetime τ_1_ of 5 ps, fluorescence lifetime τ_2_ of 20 ps, fluorescence lifetime τ_3_ of 100 ps.

#### Experiment 5: ganglion cell layer in a porcine retina sample

Autofluorescence of porcine fundus samples was measured ex vivo using two-photon excited fluorescence imaging. The technical setup was based on an inverted multi-photon laser scanning microscope (Axio Observer Z.1 and LSM 710 NLO, Carl Zeiss, Jena, Germany). A femtosecond Ti:Sapphire laser (Chameleon Ultra, Coherent Inc., Santa Clara, CA) with a pulse width of 140 fs, a pulse repetition rate of 80 MHz and a wavelength of 760 nm was used to excite the autofluorescence. To measure the time-, space- and spectrum-resolved fluorescence decay datasets (FLIM data), the technical setup for the fluorescence lifetime imaging was similar to the FLIO instrumentation. The system also splits the fluorescence photons into two spectral channels (500–560 nm and 560–700 nm). Young porcine eyes have been obtained from a local slaughterhouse and kept on ice in Dulbecco's Modified Eagle's Medium (DMEM) cell culture medium (Invitrogen, Karlsruhe, Germany) shortly after enucleation. The ocular fundus samples were taken from a paramacular region and placed into a sample holder filled with DMEM, which was placed onto the object mount of the microscope with the retina facing towards the excitation laser. Technical setup and sample preparation have been described in detail elsewhere [44]. The FLIM data were binned by the adaptive binning approach with a threshold of 10,000 photons per pixel and analyzed using a multi-exponential model (Eq 3) with two exponential functions.

## Results

### Experiment 1: Patient with Diabetes Mellitus

**Fig 7:**
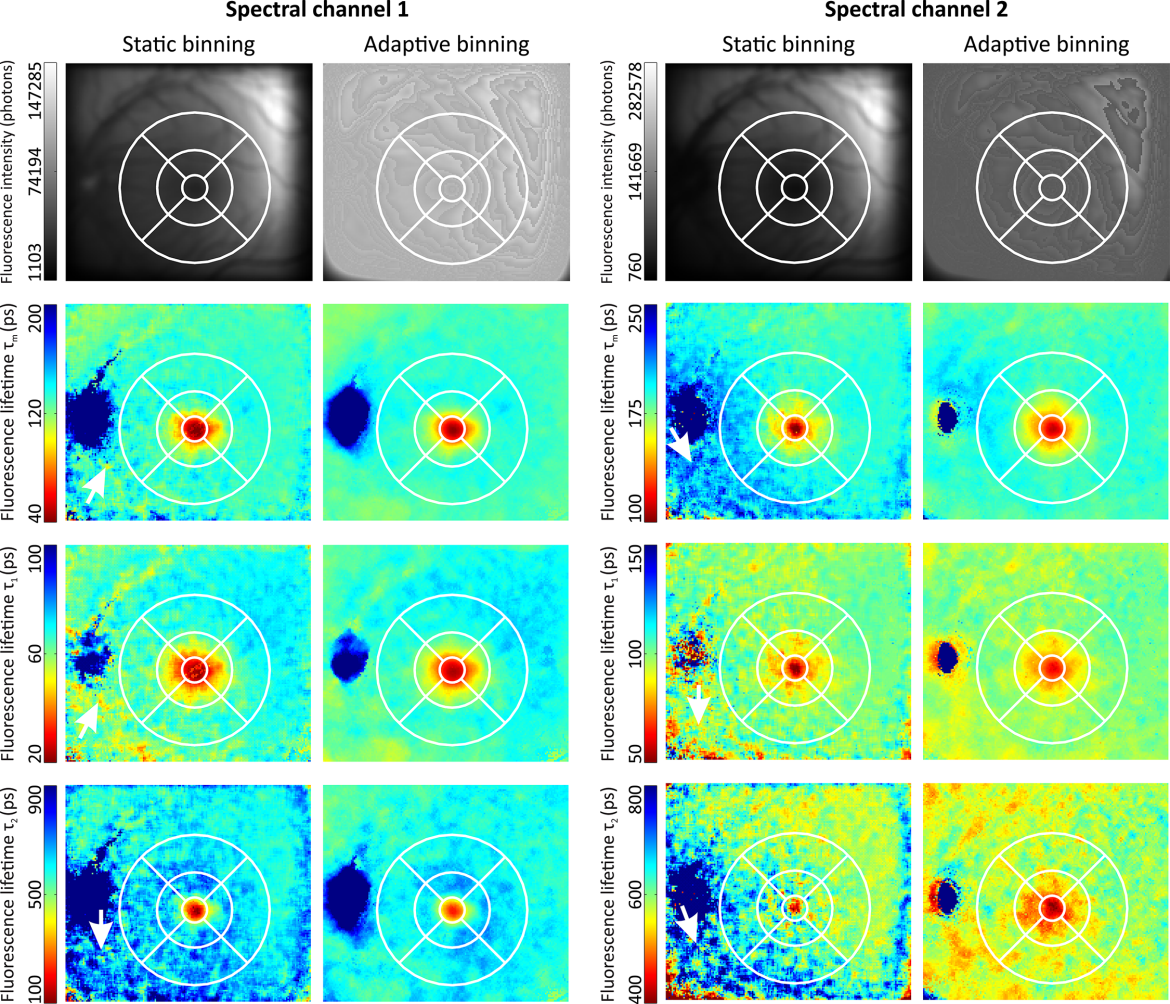
Comparison of static and adaptive binning in a diabetes mellitus patient without diabetic retinopathy. The images (149 x 169 pixels, 59 x59 μm^2^/pixel) of the fluorescence intensity, the fluorescence lifetimes τ_m_, τ_1_, and τ_2_ are shown in the rows from top to bottom. The columns are static binning and adaptive binning for both spectral channels respectively. The fluorescence lifetimes were determined using the lens-corrected approach (Eq 8), with two exponential functions, ß set to 1 and a separate crystalline lens measurement. The color scaling is identical for the fluorescence intensity and the fluorescence lifetimes in each spectral channel for better comparison. The ETRS grid is drawn on each subplot for orientation. The low amount of detected fluorescence photons in the lower left part of the image causes a prolongation of especially fluorescence lifetimes τ_2_ in both spectral channels as well as the average fluorescence lifetime τ_m_ in spectral channel 2 in case of static binning. The largest differences are highlighted by white arrows.

Fig 7 shows the fluorescence intensity of static and adaptive binning as well as the average fluorescence τ_m_ (Eq 13) and fluorescence lifetimes τ_1_, τ_2_ and τ_3_ for both spectral channels. As the patient has no diabetic retinopathy, no pathologic changes are visible in the images. The amount of detected fluorescence photons is low in the lower left part of the image. This causes in case of static binning a prolongation of especially fluorescence lifetimes τ_2_ in both spectral channels and τ_m_ in spectral channel 2. Adaptive binning shows the expected rather homogeneous fluorescence lifetimes around macular and optic disc.

### Experiment 2: Healthy Volunteer

**Fig 8:**
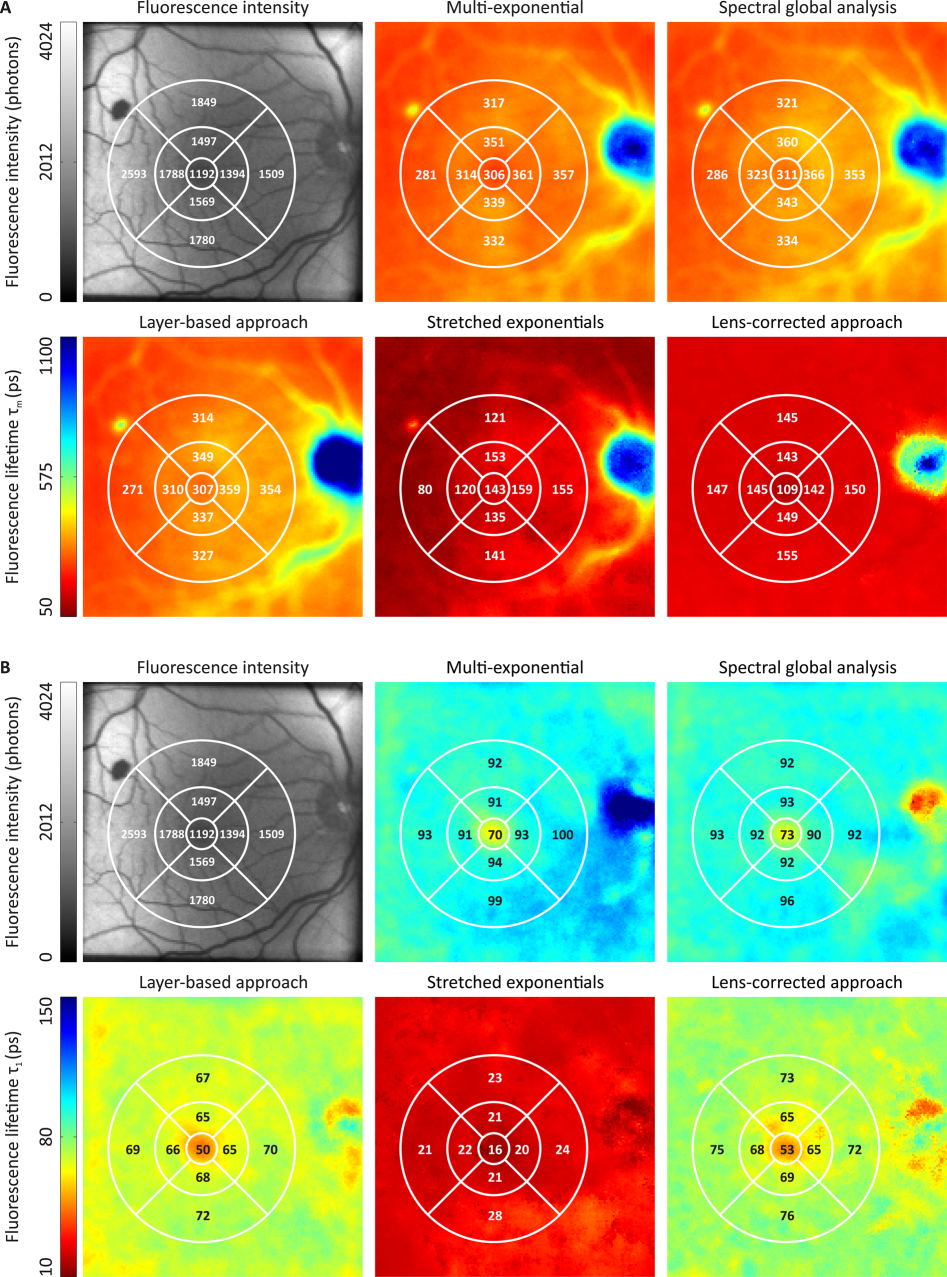
Comparison of the different fluorescent lifetime modelling approaches in a healthy volunteer. The fluorescence intensity as well as the fluorescence lifetimes τ_m_ (A) and τ_1_ (B) in spectral channel 1 are shown (154 x 154 pixels, 59 x59 μm^2^/pixel). The color scaling of the fluorescence lifetime plots is identical in each subfigure. The ETDRS grid is drawn on each subplot, and the mean values are given for each subfield of the grid. The fluorescence intensity image is the measured signal before binning.

Fig 8A depicts the fluorescence intensity as well as the average fluorescence lifetime τ_m_ (Eq 13) and fluorescence lifetime τ_1_ (Fig 8B) in spectral channel 1 for the different fluorescence lifetime approximation models. In the case of the stretched exponentials, only two exponential functions were used, compared to the three exponential functions used in the other cases. Furthermore, the fluorescence lifetime of a stretched exponential is not directly comparable to the other approaches because it must be interpreted in conjunction with the corresponding β.

The approximated average fluorescence lifetimes were relatively similar, regardless of the different models underlying the approximation. The stretched exponentials produced shorter fluorescence lifetime values in comparison to the multi-exponential, spectral global analysis and layer-based approaches but also showed very similar features, such as longer fluorescence lifetimes at the thicker blood vessels, the optic disc and the very low fluorescent spot at the upper left part of the image. The lens-corrected approach did not show such prolonged fluorescence lifetimes, except for the optic disc. Furthermore, the average fluorescence lifetimes of the lens-corrected approach were shorter and more homogenous in comparison to the multi-exponential, spectral global analysis and layer-based approaches.

The fluorescence lifetimes τ_1_, typically the exponential function with the highest amplitudes, showed larger differences. Only the multi-exponential model detected longer fluorescence lifetimes in the optic disc. Interestingly, the results of the layer-based approach and the lens-corrected approach were very similar, although their average fluorescence lifetimes showed differences, especially at the locations of the larger vessels.

### Experiment 3: Patient with Macular Hole

**Fig 9:**
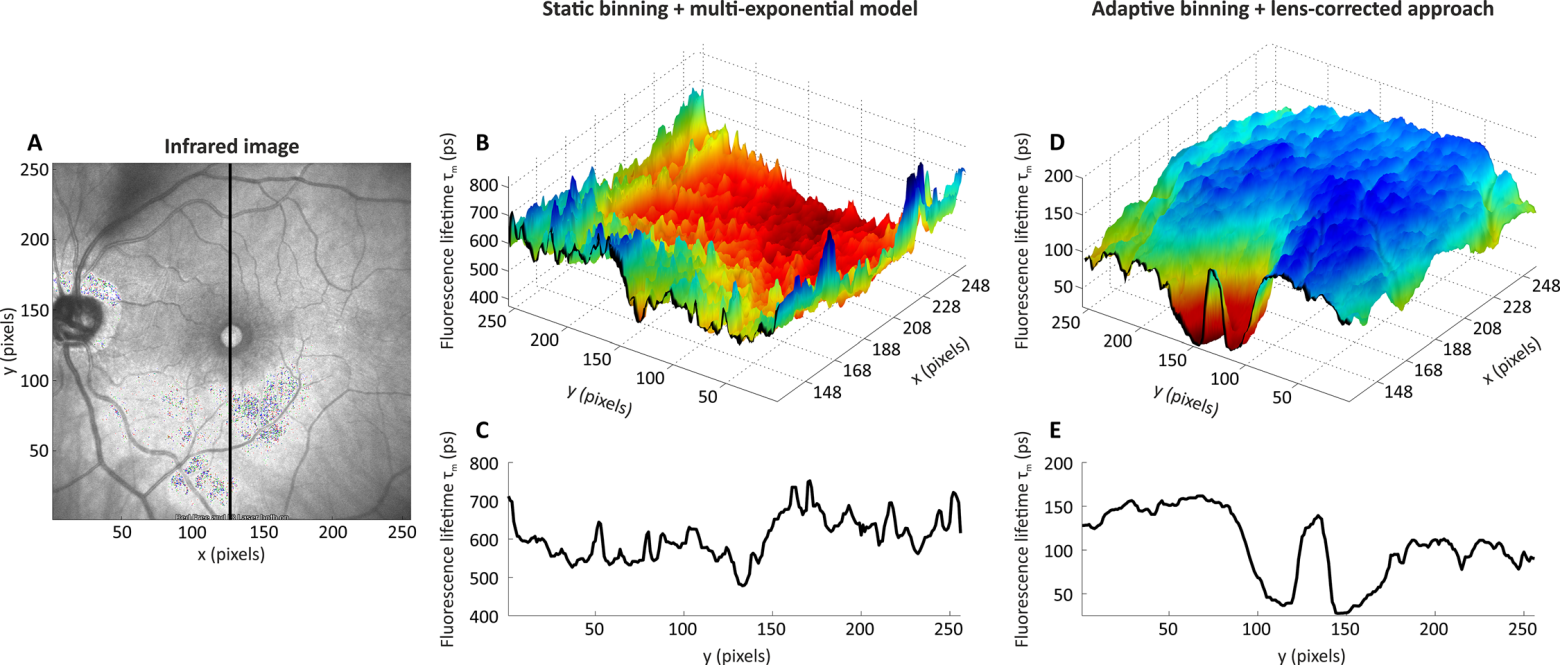
Overview of FLIMX’s visualization capabilities and comparison of static binning + multi-exponential model and adaptive binning + lens-corrected approach for a patient with macular hole. (A) shows the infrared image of the fundus (256 x 256 pixels). The FLIO data are analyzed using a multi-exponential model with three exponential functions based on static binning (factor two) and by the lens-corrected approach with two exponential functions and a separate crystalline lens measurement based on adaptive binning (threshold 100,000 photons per pixel). A vertical cross-section through the fovea centralis at pixel 128 on the x axis is highlighted as a black line. (B) and (D) show the remaining fundus section (pixels 128 to 256 on the x axis, all y pixels) in a three-dimensional view of the average fluorescence lifetime τ_m_ in spectral channel 1 for static binning + multi-exponential model and adaptive binning + lens-corrected approach respectively. The average fluorescence lifetimes τ_m_ along the cross-section are shown in detail in (C) and (E). The average fluorescence lifetimes are shorter in (D) and (E) because of the eliminated influence of the crystalline lens.

Fig 9 depicts the infrared image (Fig 9A), the average fluorescence lifetime τ_m_ obtained using static binning (factor two) and a multi-exponential model with three exponential functions (Fig 9B and 9C) as well as the average fluorescence lifetime τ_m_ obtained using adaptive binning (threshold 100,000 photons) and the lens-corrected approach with two exponential functions and a separate measurement of the crystalline lens (Fig 9D and 9E) of a patient with a macular hole. The average fluorescence lifetime is presented in a three-dimensional view (Fig 9B and 9D) beginning at a vertical cross-section through the fovea. The three-dimensional view of the average fluorescence lifetime (Fig 9B and 9D), in combination with a detailed illustration of a user defined cross-section in the image (Fig 9C and 9E), allowed for an in-depth analysis of the local changes in the patient. Cross-sections can be placed at any horizontal or vertical position of an image. Clearly, the average fluorescence lifetimes of the lens-corrected approach are much shorter in comparison to the multi-exponential model because the influence of the crystalline lens is eliminated. The missing retina tissue inside the macular hole results in a longer average fluorescence lifetime. Without lens-corrected approach, it is not possible to visualize the macular hole in the average fluorescence lifetime τ_m_.

### Experiment 4: Diabetes Patients and Healthy Controls

**Fig 10:**
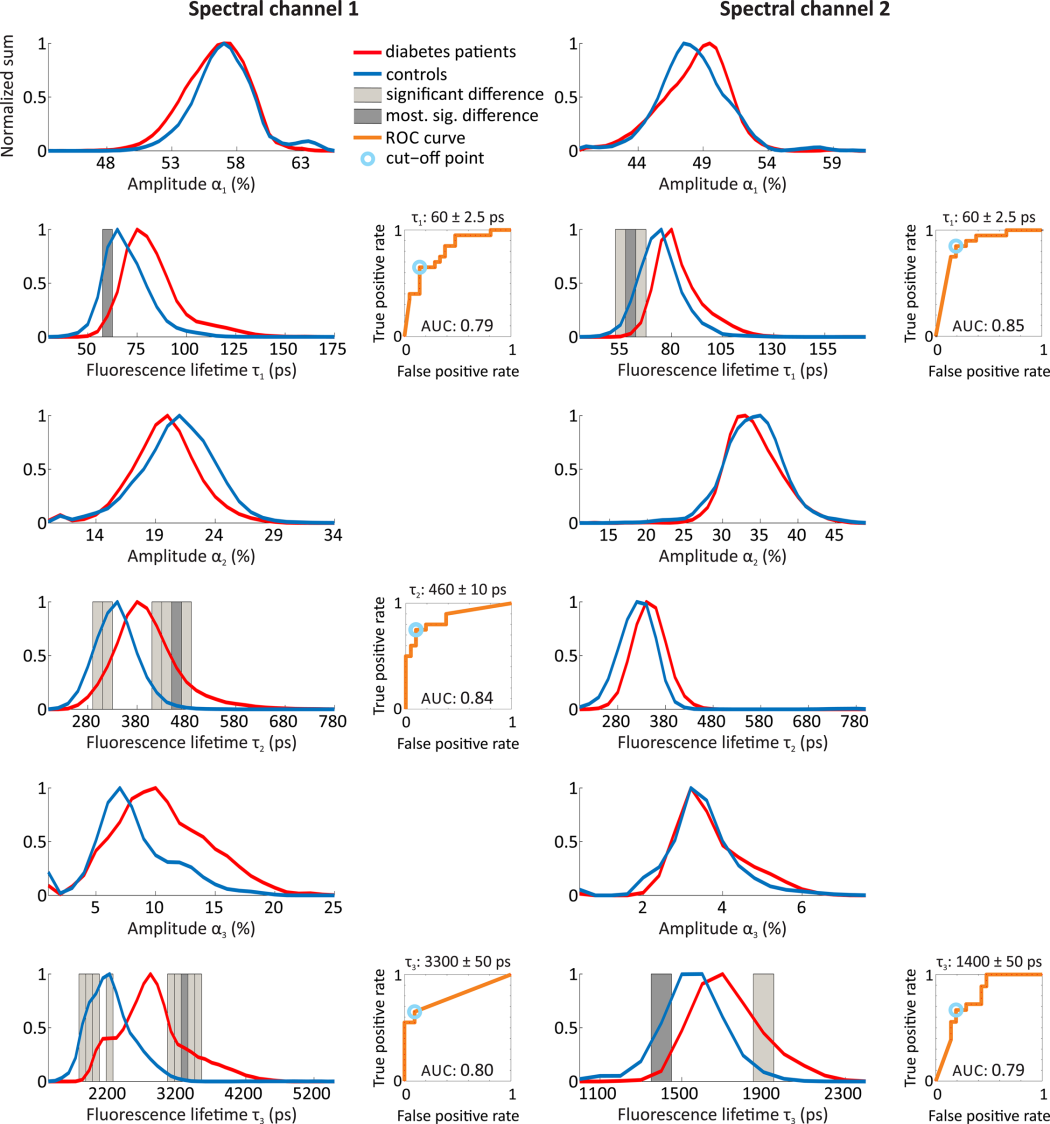
Results of the Holm-Bonferroni method applied to FLIO measurements to allow for group comparisons between diabetes patients and controls. The normalized histograms of the fluorescence amplitudes α and lifetimes τ in both spectral channels are obtained from a multi-exponential approximation using three exponential functions, for controls (blue) and diabetes patients (red). Histogram classes with significant differences, according to the Holm-Bonferroni method, are colored in light gray. The class with the highest significance level (the smallest p value) is indicated in dark gray. Only the fluorescence lifetimes showed significant differences, except for τ_2_ in spectral channel 2. For the class with the highest significance level, the corresponding receiver operating characteristic curve (orange) is shown next to the histogram. The cut-off point as best trade-offs between true positive rate and false positive rate is colored in light blue. The AUC is given under the ROC curve.

As stated above, the motivation of this experiment is to show the ability of our analysis chain to provide group comparison for groups of patients or volunteers because of the high inter-individual variability. Normalized histograms comparing the diabetes patients and controls for all of the fluorescence amplitudes and lifetimes, as well as for both spectral channels, are shown in Fig 10. A shift to longer fluorescence lifetimes in the diabetes patients is clearly visible in all fluorescence lifetimes and both spectral channels. Significantly different populated histogram classes were found for all of the fluorescence lifetimes, except for τ_2_ in spectral channel 2. No significant differences were observed for the fluorescence amplitudes. For each histogram class with the highest significance level, the corresponding receiver operating characteristic (ROC) curve is given next to the histogram. The area under the ROC curve (AUC), as a measure of the ROC curve’s accuracy, achieves the largest value of 0.85 for fluorescence lifetime τ_1_ in spectral channel 2. Thus, its cut-off point as best trade-offs between true positive rate and false positive rate would result in the best achievable diabetes detection using only the FLIO data from this experiment.

### Experiment 5: Ganglion Cell Layer in a Porcine Retina Sample

**Fig 11:**
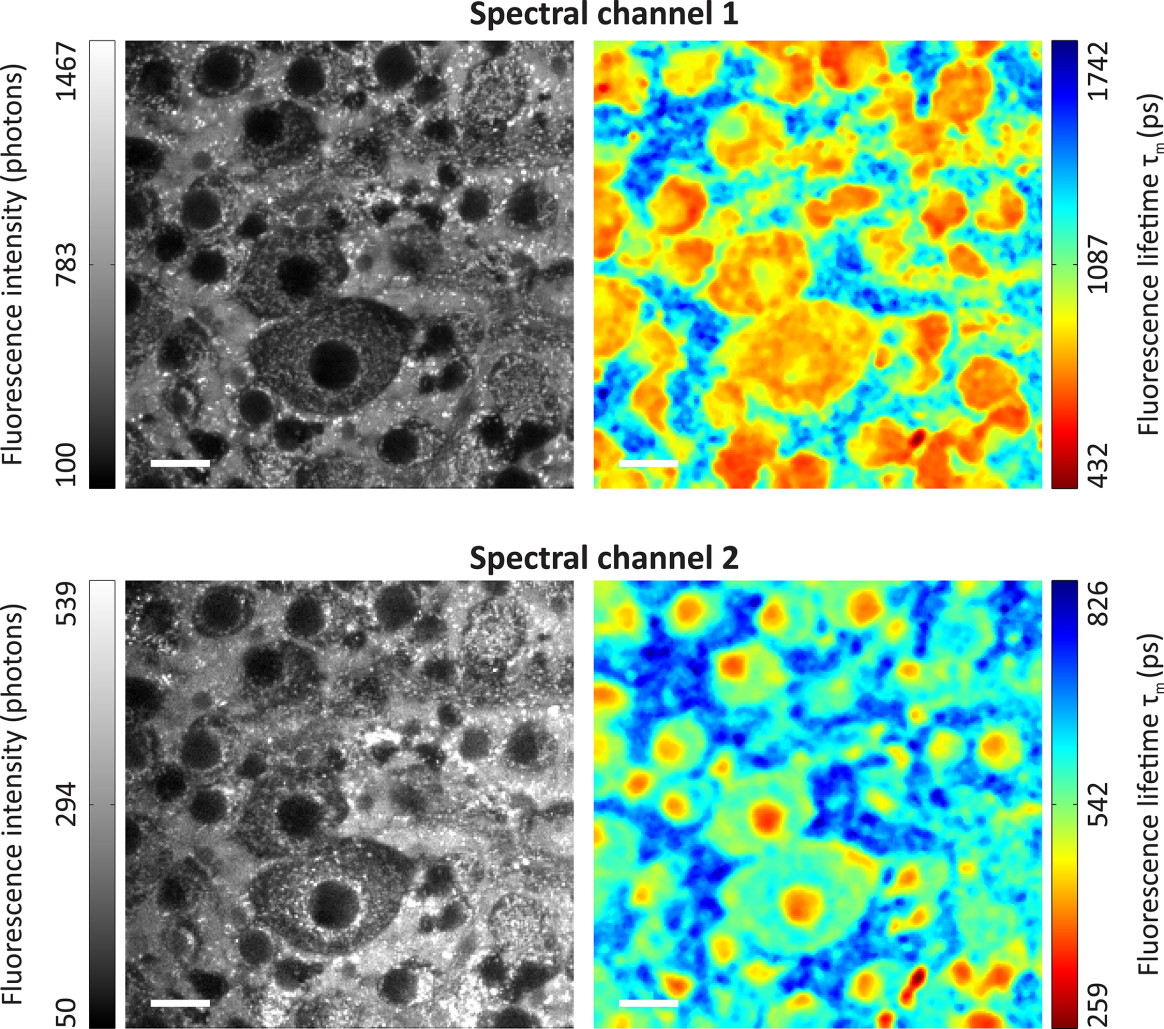
Fluorescence intensity and average fluorescence lifetime of the ganglion cell layer in a porcine retina *ex vivo* sample. The 256 x 256 pixels images (34 x34 μm^2^/pixel) of the fluorescence intensity before binning (left) and the average fluorescence lifetime τ_m_ (right) of the ganglion cell layer in a porcine retina sample are shown in two spectral channels (top: 500–560 nm; bottom: 560–700 nm). Adaptive binning with a threshold of 10,000 photons per pixel was applied. A multi-exponential model with two exponential functions was used to determine the fluorescence lifetimes. The length of the white bar is 20 μm.

The fluorescence intensity and the average fluorescence lifetime τ_m_ for spectral channels 1 and 2 are shown in Fig 11. In spectral channel 1, the ganglion cells possess much shorter average fluorescence lifetimes (700 ps–900 ps) than their surroundings. In spectral channel 2, the cell bodies also possess shorter average fluorescence lifetimes (450 ps–650 ps) than their surroundings while the cell nuclei possess the shortest average fluorescence lifetimes (350 ps–450 ps).

## Discussion

In this work, a new software package called FLIMX is introduced. This software implements a new adaptive binning method, a number of known as well as new approaches for modelling FLIO data, new methods for the treatment of artifacts and outliers, visualization and group comparison abilities specifically useful for FLIO, but generally also for other means of imaging fluorescence lifetimes. The abilities of FLIMX were demonstrated in four experiments using *in vivo* measurements from volunteers and one experiment using an *ex vivo* microscopy measurement.

Static binning smoothes all of the image parts with the same strength. In contrast, adaptive binning smoothes dark parts more and bright parts less, better preserving spatial detail. Static binning and adaptive binning also use different window shapes. Because of the circularly shaped window used in adaptive binning, only the closest pixels are used for binning. In the case of static binning, a square window shape is used, and pixels at the edges of the square shaped region are farther away from the root pixels than the other pixels. The circular window is isotropic, while the square window is anisotropic. The calculated fluorescence lifetimes in Figs 2 and 7 for raw data, static binning and adaptive binning are only of exemplary nature and are not suitable for a general comparison of other types of data.

FLIMX implements a number of known approaches [9] to model FLIO data, such as the multi-exponential approach, the stretched exponential approach, the spectral global analysis approach and incomplete decay, as well as new approaches, such as the layer-based approach, the lens-corrected approach and any combination of the mentioned approaches. A systematic investigation of which approach is best suited for FLIO is beyond the scope of this work. The layer-based approach and its extension, the lens-corrected approach, are the first methods in FLIM, to actively analyze the rising edge of the fluorescence signal. The layer-based approach allows for the separation of different fluorescent layers in a sample, if the time resolution is high enough. In principle, the layer-based approach is able to extract fluorescence information from different retina layers. The required FLIO system needs a time resolution in the order of 30 fs [29], which is not available today. Further evaluation of the layer-based approach is necessary.

The benefits of the new approaches proposed in this work are observable in the results of experiment 3, which compares the standard static binning in combination with the three-exponential approach to our adaptive binning in combination with our lens-corrected approach. These data indicate, that adaptive binning is able to preserve small structures such as the macular hole (circular shape with a diameter of circa 15 pixels), which are clearly visible in the average fluorescence lifetime in Fig 9D and 9E. The prolongation of the average fluorescence lifetime inside the macular hole is only visible using the approaches presented in this work because the average fluorescence lifetime of the standard approach is most probably dominated by the fluorescence of the crystalline lens. Further, the new approaches result in a considerably reduced noisiness of the average fluorescence lifetime, allowing for a better discrimination of the pathologic changes and thus, possibly better therapy monitoring. This statement is supported by the level of noise visible in the comparison of Fig 9B and 9D. Quantitatively, this is supported by the reduced standard deviation from 79 ps to 28 ps when applying the ETDRS grid outer ring to the data displayed in Fig 9B and 9D. FLIMX also implements different non-linear minimization algorithms to determine the fluorescence lifetimes and other non-linear model parameters. The stochastic minimization algorithms are much more robust in finding a good approximation result (fluorescence lifetime parameters) and are mostly independent of the initial solution (starting point), especially for the more complex models such as the lens-corrected approach. A disadvantage is that multiple runs on the same data may return different approximation results and not always the optimal approximation result. By increasing the number of iterations, an optimal approximation result can be guaranteed. This leads to another disadvantage: the required computational effort is 10–100 times higher in comparison to a deterministic minimization algorithm, as stated above. Thus, stochastic minimization algorithms are useful to determine an initial solution for an image and not very suitable for a pixel-wise fit.

In this work, fluorescence lifetimes are related to different fluorophores for simplification reasons. However, excited-state dynamics and potential excited-state depopulation pathways also influence the fluorescence decay. The method proposed to correct for artifacts caused by reflections in the optical pathway removes only the most important part of the artifacts. The time range of the detected artifacts is not entirely corrected, as the reflections possess an exponential decay that is as long as the entire FLIO signal. An accurate modelling of the reflections was not implemented because the anticipated benefits are rather small and the necessary increase in the complexity of the minimization algorithm may reduce the robustness of the fluorescence lifetime approximation, leading to unstable fluorescence lifetime estimates. Another method used to remove artifacts in FLIMX is the iterative algorithm for the treatment of outliers. This method is effective at rectifying errors in single pixels surrounded by presumably correct pixels. A similar effect may be achieved by median filtering the fluorescence lifetime images instead. However, median filtering can remove small fluorescence lifetime alterations, e.g., from drusen, and could introduce new artifacts into the fluorescence lifetime images.

An important step for the quantitative analysis of FLIO data is the adoption of the ETDRS grid proposed by Dysli [40] because its application is established in ophthalmology. A disadvantage is the relatively low number of pixels in each subfield, e.g., 665 pixels in the central subfield, given the spatial resolution of the current instrumentation, which may increase the variance in the statistical analysis compared to larger ROIs.

The Holm-Bonferroni method used in experiment 4 with 41 measurements, each consisting of two spectral channels, required 452 MB of memory. In this example, a single subject required approximately 11 MB of memory for the data structures in FLIMX. The amount of memory per subject may vary due to spatial resolution, ROI size and the number of spectral channels. Consequently, group comparisons in FLIMX are limited by the memory of the computer being utilized. In the era of 64 bit operating systems and common memory sizes of at least 4 GB, this limitation does not seem critical. Another computational resource is CPU time, which is especially important for the fluorescence lifetime computations. As seen in Fig 6, a distributed computation for a single measurement took approximately 17 minutes. Some available software packages are considerably faster, most likely due to lower overhead in implementations other than MATLAB, being restricted to simpler modelling approaches for the FLIO data, such as the multi-exponential approach, or the application of different minimization algorithms and parameters.

FLIMX permits the simulation of multiple decay processes, which might be helpful for interpretation of experimental data or for estimation of required number of photons [28].

## Conclusion

A new public domain software package called FLIMX has been demonstrated. This software has been optimized to extract and analyze fluorescence lifetime data from time-resolved autofluorescence measurements of the human eye, but it is not restricted to this FLIM application. FLIMX enables detailed investigations of single patients, as well as statistical analysis on groups of patients, and is most suited for FLIO research. FLIMX, is available under an open source license at http://www.flimx.de.

## Supporting Information

S1 Fig Schematic of the FLIMX software structure. The gray boxes are related to data storage, the blue boxes symbolize computational modules and the orange boxes symbolize graphical user interfaces. To reduce the complexity of the figure, only the most important connections between the modules are shown.
(EPS)

S2 Fig Schematic of the FLIMX data structure. The FLIMX data structure is divided into groups of subjects called studies. Each subject may contain multiple spectral channels. Inside a channel, the measurement data (TCSPC) and a set of corresponding results from the fluorescence lifetime approximation, such as amplitudes and lifetimes, can be saved. A channel can also contain approximation results imported from third-party software.
(EPS)
